# Supplementary material for: Iterative rolling difference-Z-score and machine learning imputation for wind turbine foundation monitoring
Source: PLoS One. 2025 Sep 5;20(9):e0331213. doi: 10.1371/journal.pone.0331213 (PMC12412959; doi:10.1371/journal.pone.0331213)
Supplement: Attachment — Submitted filename: Response to Reviewers.docx [file pone.0331213.s002.docx]

**Subject:** Response to Reviewer Comments for Manuscript PONE-D-24-34569
**Title:** Research on Anomaly Detection and Data Imputation for Wind Turbine Foundation Reinforcement Monitoring Data
**Author name:** ZhaoJizhang

***Reply to the comments***

Dear Dr. Premkumar and Reviewers ,

Thank you for the opportunity to revise our manuscript entitled *“Research on Anomaly Detection and Data Imputation for Wind Turbine Foundation Reinforcement Monitoring Data”* (PONE-D-24-34569). We greatly appreciate the constructive feedback and thoughtful suggestions provided by the reviewers. We have carefully addressed all the concerns raised, and we believe the revisions have significantly enhanced the quality of the manuscript. Below, we present a detailed response to each comment. We hope that the revised manuscript will now meet the journal's standards and be deemed suitable for publication.

**Reply to the comments of Reviewer #1:**

***Comments*:**

(1) The authors should explain the rationale behind setting the maximum number of iterations to 10 loops in the anomalous data detection process using the iterative rolling difference Z-score algorithm.

***Reply:*** Thank you for your helpful suggestion. We have provided a more detailed explanation in the "Methods" section of the revised manuscript (lines 194-230), and we have also included some recommendations for tuning the Iterative Rolling Difference Z-Score algorithm.

***Comments*:**

(2) The authors need to clarify how they adjust the Z-score threshold and the rolling difference range to identify inconsistent data, particularly in cases with substantial missing data.

***Reply:*** ***T***hank you very much for your suggestion. We have clarified in the revised manuscript (lines 194-230) how the Z-Score threshold and rolling difference range are dynamically adjusted based on the amount of missing data, ensuring the effectiveness of anomaly detection in cases with substantial missing data.

***Comments*:**

(3) In their imputation of missing data using machine learning models, the authors only employ two performance indicators: Mean Squared Error (MSE) and Mean Absolute Error (MAE). It would be beneficial to include additional statistical performance metrics, such as the Coefficient of determination, Root Mean Square Error, and Variance Account Factor.

***Reply:*** Thank you for this suggestion. We have added the requested performance metric: Root Mean Squared Error (RMSE). The updated results, incorporating this additional metric, can be found in the revised manuscript (lines 283 and 312).

***Comments*:**

(4) The number of datasets used for machine learning training and testing should be specified in the text, along with a validation of the results included in the report.

***Reply:*** We appreciate this important suggestion. We have now included the number of datasets used for training and testing in the revised manuscript (lines 323-324).

***Comments*:**

(5) The axis titles in Figure 11 are not visible and require improvement. Additionally, Figure 15 also needs enhancements.

***Reply:*** Thank you for pointing this out. We have revised Figures 11 and 15 to enhance the visibility of the axis titles, as well as made other necessary improvements to ensure the clarity of the figures, in line with the reviewer’s suggestions.

***Comments*:**

(6) In line 265, the phrase “the results are presented in the table below” should specify the table number instead of using such a general reference.

***Reply:*** Thank you for this suggestion. We agree with your recommendation and have updated the manuscript accordingly. The phrase "the results are presented in the table below" has been replaced. The revised manuscript now reads: "The results are presented in Table 3 below, and Figure 10 clearly indicates that the method proposed in this study demonstrates excellent imputation performance for missing data, highlighting the feasibility and effectiveness of the approach" (lines 306-309).

***Comments*:**

(7) Please correct the typographical errors in line 245 ("subpar") and line 250 ("efficacy").

***Reply:*** We sincerely apologize for the typographical errors. These have been corrected in the revised manuscript as follows:

a) In this experiment, traditional Support Vector Machine (SVM) and Ridge Regression exhibited poor performance in capturing the complex relationships within the data.(lines 286-287)
b) To validate the effectiveness of linear interpolation in addressing isolated missing data, ...(lines 292)

**Reply to the comments of Reviewer #2:**

***Comments*:**

**(1)** The literature is insufficient. Related past studies mainly from the last 5 years should be added to claim the novelty.

***Reply:*** Thank you for your valuable feedback. We have reviewed and incorporated relevant studies from the past five years to strengthen the literature review and highlight the novelty of our approach. The updated references can be found in the revised manuscript (lines 463-480, 510-516).

***Comments*:**

(2) The text in most of the figures is not clearly visible

***Reply:*** We appreciate your feedback. We have improved the font size and clarity of the text in all figures to ensure they are now legible and meet PLOS ONE’s publication standards. The revised figures have been included in the manuscript.

***Comments*:**

(3) Authors have claimed that the applied method is well-suited for the detection and cleansing of anomalies in time-series data. The statement should be supported with reference to similar past studies.

***Reply:*** We appreciate your suggestion and have added references to relevant past studies that support the effectiveness of our method for anomaly detection and cleansing in time-series data. These references can be found in the updated manuscript (lines 53-70).

***Comments*:**

(4) The Conclusion section should start with a paragraph describing a brief recap of the work followed by the conclusive statements.

***Reply:*** Thank you for your suggestion. We have revised the Conclusion section to begin with a brief recap of the work, followed by the conclusive statements, as recommended. This revision aims to provide a clearer and more structured summary of our findings.

***Comments*:**

(5) The conclusions are too limited. As per the performed work having multiple aspects, the conclusions should elaborate on the detailed dominating results to emphasize the efficacy of the adopted method.

***Reply:*** We appreciate your valuable feedback. We have expanded the Conclusion section to provide a more detailed discussion of the key findings and their implications. Additionally, we have emphasized the efficacy of the adopted method in addressing the challenges of anomaly detection and data imputation in wind turbine monitoring.
