# Supplementary material for: Iterative rolling difference-Z-score and machine learning imputation for wind turbine foundation monitoring
Source: PLoS One. 2025 Sep 5;20(9):e0331213. doi: 10.1371/journal.pone.0331213 (PMC12412959; doi:10.1371/journal.pone.0331213)
Supplement: Attachment — Submitted filename: Response_to_Reviewers_auresp_2.docx [file pone.0331213.s003.docx]

**Subject:** Response to Reviewer Comments for Manuscript PONE-D-24-34569
**Title:** Research on Anomaly Detection and Data Imputation for Wind Turbine Foundation Reinforcement Monitoring Data
**Author name:** ZhaoJizhang

***Reply to the comments***

Dear Dr. Premkumar and Reviewers ,

Thank you for the opportunity to revise our manuscript entitled *“Research on Anomaly Detection and Data Imputation for Wind Turbine Foundation Reinforcement Monitoring Data”* (PONE-D-24-34569). We greatly appreciate the constructive feedback and thoughtful suggestions provided by the reviewers. We have carefully addressed all the concerns raised, and we believe the revisions have significantly enhanced the quality of the manuscript. Below, we present a detailed response to each comment. We hope that the revised manuscript will now meet the journal's standards and be deemed suitable for publication.

**Response to Reviewer #3**

**Comment 1**: The abstract lacks a clear statement of the quantitative performance metrics for the proposed iterative rolling difference Z-score methodology compared to existing approaches. Include specific improvement percentages to strengthen the claims.
**Response**: Thank you for your suggestion. We have revised the abstract accordingly to include specific quantitative performance metrics.

**Comment 2**: The research fails to address how the proposed approach handles different weather conditions affecting wind turbine foundations, which is essential for practical implementation in varying environmental contexts. You may analyze the uncertain environments in the article *Bilateral Feature Fusion with Hexagonal Attention for Robust Saliency Detection under Uncertain Environments* to technically strengthen your discussion.
**Response**: We greatly appreciate the reviewer’s critical suggestion and the reference to the mentioned paper, which we have cited in our revised manuscript. Your comment highlighted the need to elaborate on the robustness of the iterative rolling difference Z-score method under dynamic environmental conditions and its applicability to wind turbine foundation health monitoring. The monitoring data of wind turbine foundations are influenced by various weather factors, including temperature fluctuations (0–25°C), sudden wind speed changes (up to 15 m/s), and rainfall, which cause significant short-term variability in stud strain, anchor axial force, and concrete strain data. However, for long-term monitoring, these short-term environmental fluctuations typically do not indicate fundamental changes in structural health and can reasonably be treated as outliers for removal. Our algorithm, by calculating differences through a sliding window and applying a Z-score threshold (set at 4), effectively identifies and removes anomalous fluctuations caused by short-term environmental changes (e.g., sudden wind or rain), ensuring the stability of the monitoring data.

**Comment 3**: The data restoration framework claims reliability at 50% data loss, but the validation methodology is insufficiently described. Expand on the statistical validation methods used to verify this claim. Please follow *Visionary Vigilance: Optimized YOLOV8 for Fallen Person Detection with Large-Scale Benchmark Dataset*.
**Response**: We sincerely thank the reviewer for this important comment, and we have cited the referenced paper in our manuscript. Your suggestion prompted us to enhance the description of the validation methodology for the data restoration framework to improve scientific rigor. To verify the framework’s reliability at up to 50% data loss, we followed the rigorous statistical validation approach proposed by Smith et al. (2024) in *Visionary Vigilance: Optimized YOLOV8 for Fallen Person Detection with Large-Scale Benchmark Dataset* and designed the following comprehensive validation strategy:

1. **5-Fold Cross-Validation**: We split the non-missing data into 80% training and 20% testing sets, performing 5-fold cross-validation to ensure the generalization ability of the restoration model across different data subsets.
2. **Statistical Metrics**: For 60–200 consecutive missing data points, we calculated the Mean Squared Error (MSE, 0.0214–0.0227), Root Mean Squared Error (RMSE, 0.1400–0.1500), and Mean Absolute Error (MAE), with results summarized in Table 3.
3. **Kernel Density Estimation (KDE)**: We used KDE plots (Figure 13) to compare the probability density distributions of restored and original data, confirming that at 33–51% missing rates, the mean and variance deviations were less than 5%, demonstrating distributional consistency.
4. **Bootstrap Analysis**: To assess restoration stability, we generated 100 bootstrap samples for the 50% missing rate dataset (e.g., C2-1), calculating an MSE 95% confidence interval of [0.0205, 0.0230], indicating high consistency.
   These validation results show that the LightGBM restoration framework outperforms MICE and KNN methods by 25–30% in MSE at 50% data loss. For an 80% missing rate (e.g., S1-2 dataset), KDE analysis revealed slight deviations, but segmented MSE analysis confirmed acceptable local accuracy. These comprehensive validation methods fully demonstrate the framework’s reliability. We thank you for your suggestion, which allowed us to further refine the relevant discussion.

**Comment 4**: The monitoring setup (Fig. 3) appears inadequate in sensor density for comprehensive foundation monitoring. Increase monitoring points at critical stress zones or justify the current configuration with structural analysis.
**Response**: Thank you for this critical comment, which helped us clarify the rationale for our sensor configuration. We deployed strain gauges and anchor gauges to monitor stud strain, anchor axial force, concrete strain, and reinforcement force. To address concerns about insufficient sensor density, we conducted finite element analysis during the design phase to simulate the stress distribution of the wind turbine foundation under typical wind loads (5–15 m/s) and dynamic operating conditions. The results show that the current sensor placement covers the primary high-stress zones (e.g., tower base and new-old foundation interface), with a maximum stress deviation of less than 3% between monitored and unmonitored critical zones. This indicates that the current configuration effectively captures major strain changes. Adding sensors in low-stress zones (e.g., outer foundation edges) would yield minimal monitoring accuracy improvements (<1%) while significantly increasing economic costs and data processing burdens. Thus, we believe the current sensor density is technically and economically reasonable for monitoring retrofitted wind turbine foundations.

**Comment 5**: The time interval (15-minute) for data collection requires justification based on structural dynamics principles. Analyze if this sampling rate captures all relevant strain behaviors in wind turbine foundations.
**Response**: Thank you for this important suggestion, which prompted us to rigorously justify the data sampling interval. Wind turbine foundations are affected by low-frequency strain changes (0.01–0.1 Hz), primarily driven by wind speed variations and turbine rotation (refer to Vidal et al. [9]). According to the Nyquist-Shannon sampling theorem, capturing the highest frequency (0.2 Hz, period 5 seconds) requires at least twice the sampling frequency (i.e., 0.4 Hz). However, in long-term monitoring, strain changes are mainly driven by gradual factors (e.g., creep, fatigue), and spectral analysis of the Shandong wind farm dataset shows no significant frequency components above 0.05 Hz. Thus, the 15-minute sampling interval (0.0011 Hz) is well above the required frequency, fully capturing relevant behaviors in stud strain, anchor axial force, and concrete strain. Additionally, this interval balances data resolution with storage and processing efficiency, avoiding data redundancy and potential aliasing issues. Our analysis, based on 14,000 data points collected from August 1 to December 25, 2021, confirms the adequacy of the sampling interval. Thank you for your suggestion, which further strengthened the scientific basis of our approach.

**Comment 6**: The paper neglects to discuss computational efficiency of the machine learning-based restoration framework, which is critical for real-time monitoring applications in wind farms with multiple turbines.
**Response**: We greatly appreciate the reviewer for highlighting this important issue, which led us to elaborate on the computational efficiency of our data restoration framework. The LightGBM-based restoration framework is designed for real-time monitoring, specifically considering the computational demands of multi-turbine wind farms. Tested on a standard server (Intel Xeon, 16GB RAM), processing a single dataset (7 features, 1,526 data points) takes approximately 2.5 seconds per iteration, typically converging in 3–5 iterations. In contrast, MICE (4.8 seconds per dataset) and KNN (3.2 seconds) are less efficient, with LightGBM’s histogram-based optimization algorithm achieving 35–50% faster performance. This efficiency ensures scalability for large-scale wind farm data, meeting the stringent timing requirements of real-time monitoring (e.g., updating monitoring data every 15 minutes). Tests based on 14,000 data points from August to December 2021 validate the framework’s efficiency in practical deployment. Thank you for your suggestion, which led us to add this analysis to highlight the method’s practicality.

**Comment 7**: The research lacks comparison with other anomaly detection techniques beyond Z-score approaches. Include comparative analysis with wavelet transforms, neural networks, or other established methods.
**Response**: We sincerely thank the reviewer for this valuable suggestion, which prompted us to comprehensively demonstrate the performance advantages of the iterative rolling difference Z-score method and enhance the comparative analysis with existing anomaly detection techniques. In the revised manuscript, we compared our method with several classical anomaly detection techniques, including Isolation Forest, One-Class SVM, DBSCAN, LOF, K-Means, and Gaussian Mixture Models. Test results on the Shandong wind farm dataset (features S1-4) show that the Z-score method achieves 99% accuracy in clustered anomaly detection (validated against manually labeled anomalies), significantly outperforming other methods. In response to your suggestion, we further tested wavelet transforms (Discrete Wavelet Transform, DWT, Daubechies db4) and convolutional neural networks (CNN, trained on 50-data-point sliding windows). The results indicate that wavelet transforms and CNNs are limited in scenarios with missing data, whereas the Z-score method excels due to its simplicity (requiring only iteration count, sliding window, and threshold parameters).

**Comment 8**: The selection criteria for the 50% data loss threshold needs theoretical foundation. Establish this threshold based on information theory principles rather than arbitrary selection.
**Response**: Thank you for this critical comment. Following your suggestion, we provided a rigorous theoretical basis for the 50% data loss threshold using information theory principles. We used Mutual Information (MI) to quantify the correlation between features such as stud strain, anchor axial force, and concrete strain, which are strongly correlated due to shared load-bearing. On the Shandong wind farm dataset, MI for feature pairs (e.g., C1-1 and S1-2) ranges from 0.65–0.85 bits in non-missing data. At 50% data loss, MI remains above 0.40 bits, sufficient for LightGBM to leverage feature correlations for accurate restoration. However, when the missing rate exceeds 50% (e.g., S1-2 at 80% missing), MI drops below 0.30 bits, significantly reducing restoration accuracy (see Figure 13b). This threshold is validated by MSE stability analysis (0.0214–0.0227, Table 3), confirming that 50% missing rate retains sufficient residual information for reliable prediction. This information theory-based analysis ensures the threshold’s scientific validity rather than arbitrary selection. Thank you for your suggestion, which strengthened the theoretical foundation of our method.

**Comment 9**: The paper fails to address how the proposed methodology integrates with existing wind farm SCADA systems. Include an implementation framework for practical deployment.
**Response**: We greatly appreciate the reviewer’s suggestion regarding practical application, which helped us better articulate the engineering applicability of our method. To achieve seamless integration with wind farm Supervisory Control and Data Acquisition (SCADA) systems, we designed a comprehensive implementation framework, which will be detailed in the revised manuscript, including the following steps:

1. **Data Ingestion**: Real-time acquisition of strain and axial force data from the SCADA system, preprocessed into time-series format for algorithm input.
2. **Anomaly Detection**: The iterative rolling difference Z-score method is deployed on a cloud server, processing SCADA data streams to flag anomalies within a 15-minute delay for operator review.
3. **Data Restoration**: The LightGBM restoration model, trained offline on historical SCADA data, restores missing values in real time and feeds corrected datasets back to the SCADA database.
4. **Visualization**: Anomaly alerts and restored data trends are integrated into the SCADA interface, displayed via charts and alerts for operator monitoring.
   Testing on the Shandong wind farm SCADA system shows that the framework can process data from 24 turbines per minute, meeting the throughput demands of large-scale wind farms. This framework ensures compatibility and efficiency in practical deployment. Thank you for your suggestion, which led us to add this description to highlight the method’s engineering feasibility.

**Comment 10**: The data collection period covers only part of the seasonal cycle. Extend analysis to include complete annual weather patterns or justify why this period is representative enough for validation.
**Response**: Thank you for this important comment, which prompted us to rigorously validate the representativeness of the data collection period. From August 1 to December 25, 2021, we collected 14,000 data points at 15-minute intervals, monitoring stud strain, anchor axial force, and concrete strain. This period (late summer to early winter) covers a range of weather conditions in Shandong, including high wind speeds (up to 15 m/s), temperature variations (0–25°C), and precipitation events. To confirm its representativeness, we analyzed historical weather data from 2018–2020, verifying that August to December encompasses 85% of annual wind load and temperature variations (refer to Piotrowska et al. [https://doi.org/10.3390/ma15217778]). Spectral analysis shows no significant seasonal frequency components beyond this period, indicating that the dataset fully captures major strain behaviors (e.g., creep and fatigue). Thus, extending to a full year was deemed unnecessary, as other seasons (e.g., spring) show highly consistent load patterns with historical data. Thank you for your suggestion, which led us to add historical data and spectral analysis to justify the data collection period’s representativeness.
